# Is there a causal relationship between executive function and liability to mental health and substance use? A Mendelian randomization approach

**DOI:** 10.1098/rsos.220631

**Published:** 2022-12-14

**Authors:** Sabrina M. I. Burton, Hannah M. Sallis, Alexander S. Hatoum, Marcus R. Munafò, Zoe E. Reed

**Affiliations:** ^1^ School of Psychological Science, University of Bristol, Bristol BS8 1TH, UK; ^2^ Centre for Academic Mental Health, Population Health Sciences, University of Bristol, Bristol BS8 1TH, UK; ^3^ MRC Integrative Epidemiology Unit at the University of Bristol, Bristol BS8 2BN, UK; ^4^ Department of Psychiatry, Washington University School of Medicine, St Louis, MO 63110, USA; ^5^ National Institute for Health Research Bristol Biomedical Research Centre, University Hospitals Bristol NHS Foundation Trust and University of Bristol, Bristol BS28 2BN, UK

**Keywords:** Mendelian randomization, executive function, mental health, substance use

## Abstract

Poorer performance in tasks testing executive function (EF) is associated with a range of psychopathologies such as schizophrenia, major depressive disorder (MDD) and anxiety, as well as smoking and alcohol consumption. We used two-sample bidirectional Mendelian randomization to examine whether these may reflect causal relationships and the direction of causation. We used genome-wide association study summary data (*N* = 17 310 to 848 460) for a common EF factor score (cEF), schizophrenia, MDD, anxiety, smoking initiation, alcohol consumption, alcohol dependence and cannabis use disorder (CUD). We found evidence of increased cEF on reduced schizophrenia liability (OR = 0.10; CI: 0.05 to 0.19; *p*-value = 3.43 × 10^−12^), MDD liability (OR = 0.52; CI: 0.38 to 0.72; *p*-value = 5.23 × 10^−05^), drinks per week (*β* = –0.06; CI: –0.10 to −0.02; *p*-value = 0.003) and CUD liability (OR = 0.27; CI: 0.12 to 0.61; *p*-value = 1.58 × 10^−03^). We also found evidence of increased schizophrenia liability (*β* = −0.04; CI: −0.04 to −0.03; *p*-value = 3.25 × 10^−27^) and smoking initiation on decreased cEF (*β* = −0.06; CI: −0.09 to −0.03; *p*-value = 6.11 × 10^−05^). Our results indicate potential causal relationships between cEF and mental health and substance use. Further studies are required to improve our understanding of the underlying mechanisms of these effects, but our results suggest that EF may be a promising intervention target for mental health and substance use.

## 1. Introduction

The ability to perform nearly all of the activities required for daily living is mediated by executive function (EF) [1]—the ability to perform self-directed behaviour toward a goal and to enable self-regulation. The prefrontal cortex is one of the main neural substrates of EF, including cognitive control functions that regulate lower-level processes such as decision making [1]. There are different aspects of EF, including inhibitory control, working memory and task switching [2].

EF plays a role in many behaviours that are disrupted in a range of mental health conditions [1], and there is evidence that it is also associated with substance use [3]. For example, poorer EF has been observed among individuals with schizophrenia [1,4–6], major depressive disorder (MDD) [7,8], anxiety [9,10], as well as in people who smoke both cigarettes [11,12] and cannabis [13,14] and consume alcohol [11,15]. Mental health and substance use may be associated with EF as EF drives and directs thought processes, effortful control and emotion regulation [16], including the development and maintenance of strategies to cope with maladaptive behaviour. EF can also influence maladaptive thought processes that can manifest uniquely across differing psychiatric conditions. For example, maladaptive thought processes could lead to negative biases and social difficulties in depression [17] or thoughts of anticipation in substance use disorders [18]. Despite these possible mechanisms, the direction of association between EF and these phenotypes is still unclear [1], with some studies suggesting EF deficits prior to these [3] and others suggesting they occur after [4]. It is unclear whether these associations represent causal pathways, and if so, what the direction of any causal effect might be. EF is potentially modifiable [19], and drug repurposing analyses for a common EF factor (cEF) score, which captures variance shared across several EF tasks [20], have suggested that this cEF may also be modifiable. Therefore, if we can better understand the relationship between EF and mental health and substance use outcomes, this will help to inform intervention development.

Mendelian randomization (MR) is a well-established method for causal inference, which relies on approximations of Mendel's laws of segregation and random assortment [21]. MR is based on instrumental variable (IV) analysis, with single-nucleotide polymorphisms (SNPs) that are robustly associated with the exposure used as IVs. MR is subject to three core assumptions: (i) the genetic instrument is robustly associated with the exposure of interest (relevance), (ii) there is no confounding of the genetic instrument and the outcome (independence), and (iii) the genetic instrument only influences the outcome via the exposure (exclusion restriction). There are different MR methods that test potential violations of these assumptions and, therefore, a consistent effect estimate across different approaches would provide greater evidence of a truly causal effect, robust to the assumptions of MR. MR minimizes the effect of confounding variables as the genetic variants are randomly assigned at conception [22]. It also overcomes issues around reverse causation as these genetic variants precede any outcomes [23].

Summary data from genome-wide association studies (GWAS) can be used as the genetic instruments in MR. A recent GWAS of cEF score, conducted in the UK Biobank (European ancestry), identified 90 genome-wide significant hits [20]. The cEF factor score was created from five different EF tasks—trail-making, symbol-digit substitution, digit span, prospective memory and pairs memory—using confirmatory factor analysis. Unlike previous studies focusing on specific EF tasks, the cEF incorporates multiple facets which may better capture the cognitive component of psychopathology. In particular, single EF tasks are noisy measures of EF, with large method variance components reflecting lower-level cognitive processes (the ‘task impurity problem’ [24]). By combining multiple tasks, we can create a more ‘pure’ measure of EF. Many of the tasks included in this measure are related to working memory; however, in the original GWAS, Hatoum *et al*. [20] found that this did not bias the model to better predict working memory over variance common across all EF tasks, and that a genetic risk score of this measure predicted cEF to a greater degree than working memory, suggesting it is in fact capturing a broader measure of EF than just working memory. The trail-making task is commonly used to measure EF, and the other tasks, while less commonly used, capture other aspects. Thus, when combined for the cEF factor score, this is similar to cEF factors used in much smaller studies, and the authors conducted analyses which validated the use of this score. In addition, each EF task alone is a combination of true variance measuring cognitive direction of thought, lower-level non-EF processes such as sensory processes and measurement variance. When combined, like in the cEF factor score, this distils the common variance across tasks and better identifies EF variance against lower-level processes [24]. In line with this, Hatoum *et al*. [20] reported nearly twice the prediction from the polygenic score of cEF predicting cEF than a previous study examining only the trail-making task in the UK Biobank and prediction in the CHARGE consortium [25]. Further, past work has shown that this common component is correlated with—but separable from—IQ, and is genetically associated with psychopathology over and above the genetic influence of other cognitive factors [20].

We examined whether there were causal relationships between EF and a range of mental health and substance use phenotypes, and the direction of any effect—for example, does poor mental health lead to poorer EF, or vice versa? We did this by applying a two-sample MR approach, where the SNP-exposure and SNP-outcome estimates are obtained from GWAS in independent samples and used to estimate causal effects. We focused on schizophrenia, MDD, anxiety, smoking initiation, alcohol consumption (drinks per week), alcohol dependence and cannabis use disorder (CUD), using a bidirectional approach to determine the causal direction of these relationships.

## 2. Methods

### 2.1. Data sources

We used GWAS data from several studies, shown in table 1. To minimize sample overlap, we excluded some samples that contributed to the original GWAS in our analyses, as indicated in table 1.

**Table 1.** GWAS for executive function, mental health and substance use outcomes.

phenotype	author	sample	final *N*
executive function	Hatoum *et al*. [20]	UKBB	427 037
schizophrenia	schizophrenia working group of the PGC [26]	PGC	cases = 69 369
			controls = 236 642
MDD	Wray *et al*. [27]	PGC, excluding UKBB and 23andMe	cases = 45 396
			controls = 97 250
anxiety	Otowa *et al*. [28]	ANGST	cases = 5712
			controls = 11 598
smoking initiation	Liu *et al*. [29]	GSCAN, excluding UKBB	848 460
drinks per week	Liu *et al*. [29]	GSCAN, excluding UKBB	630 154
alcohol dependence	Walters *et al*. [30]	PGC substance use disorders working group	cases = 8485
			controls = 20 272
cannabis use disorder	Johnson *et al*. [31]	PGC substance use disorders working group, iPSYCH and deCODE	cases = 14 080
			controls = 343 736

UKBB = UK Biobank, PGC = Psychiatric Genomics Consortium, ANGST = Anxiety NeuroGenetics Study, GSCAN = GWAS and Sequencing Consortium of Alcohol and Nicotine use.

#### 2.1.1. Executive function

We used summary data from the most recent GWAS of cEF [20], which identified 90 independent genome-wide significant (*p* < 5 × 10^−08^) SNPs associated with a cEF score, where a higher score reflects increased EF.

#### 2.1.2. Schizophrenia

We used summary data from the most recent Psychiatric Genomics Consortium (PGC) GWAS of schizophrenia [26], which identified 294 independent genome-wide significant SNPs. Cases mostly included participants diagnosed with schizophrenia (although diagnoses of other psychotic disorders were also included in some samples).

#### 2.1.3. Major depressive disorder

We used summary data from the most recent PGC GWAS of MDD [27], which identified 44 independent genome-wide significant SNPs. Cases of MDD were either diagnosed by a clinical professional, or through structured interviews with trained interviewers, using the DSM-IV, ICD-9 or ICD-10 criteria.

#### 2.1.4. Anxiety

We used summary data from a GWAS of anxiety [28], which identified one independent genome-wide significant SNP. In this meta-analysis, there were up to five different anxiety disorder phenotypes included in the nine samples from seven independent cohorts. Lifetime DSM-based anxiety disorder diagnostic assessments were available for all cohorts except for the Rotterdam study, in which only 1-year prevalence was assessed. Each study assessed DSM-based criteria for the following six lifetime clinical phenotypes: generalized anxiety disorder, panic disorder, agoraphobia, social phobia, specific phobia and MDD; however, any subject reporting a mood disorder only was removed from analyses.

#### 2.1.5. Smoking initiation

We used summary data from the most recent GWAS of smoking initiation [29], which identified 378 conditionally independent genome-wide significant SNPs associated with ever being a regular smoker (current or former). Participants were asked whether they had smoked more than 100 cigarettes in their lifetime and whether they had ever smoked every day for at least a month or ever smoked regularly. To obtain summary statistics for the full sample included in the GWAS and Sequencing Consortium of Alcohol and Nicotine use (GSCAN) GWAS excluding UK Biobank data, we meta-analysed results from GWAS of 23andMe, Inc. only data and all results excluding UK Biobank and 23andMe. The meta-analysis was conducted using the genome-wide association meta-analysis software [32].

#### 2.1.6. Drinks per week

We used summary data from the most recent GWAS of drinks per week [29], which identified 99 conditionally independent genome-wide significant SNPs associated with the average number of drinks a participant reported drinking each week. Participants were asked about the number of alcoholic beverages they had in the past week and the average number of drinks per week that they had in the past year. Data were log-transformed prior to the GWAS. This measure did not account for the type of alcohol consumed, and for any study with ranges, the mid-range value was used. Again, to obtain summary statistics for the full GSCAN GWAS excluding UK Biobank data, we meta-analysed results from GWAS of 23andMe only data and all results excluding UK Biobank and 23andMe.

#### 2.1.7. Alcohol dependence

We used summary data from the PGC substance use disorders working group GWAS of alcohol dependence [30], which identified one conditionally independent genome-wide significant SNP in their European GWAS associated with alcohol dependence. Alcohol dependence cases were those that met criteria for DSM-IV or DSM-III-R alcohol dependence diagnosis.

#### 2.1.8. Cannabis use disorder

We used summary data from the most recent GWAS of CUD [31] from the PGC substance use disorders working group, iPSYCH and deCODE which identified two conditionally independent genome-wide significant SNPs associated with CUD. CUD cases were those that met criteria for DSM-5, DSM-IV, DSM-III-R or ICD-10 cannabis abuse or dependence.

### 2.2. Statistical analyses

**Figure 1.**
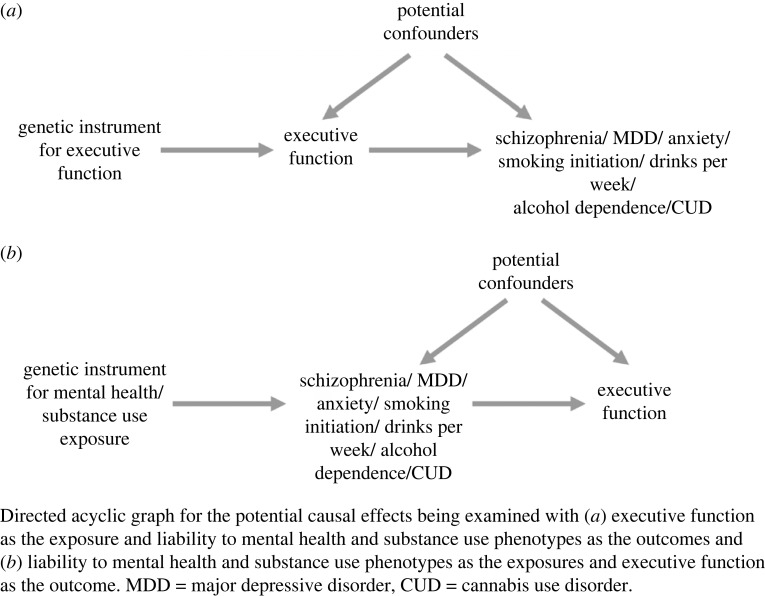
Bidirectional two-sample Mendelian randomization between a common cEF and liability to mental health and substance use outcomes.

The analysis plan for this study was pre-registered on the Open Science Framework (https://osf.io/j3tb5). We conducted two-sample MR analyses using R (v. 4.0.3) [33] and the package TwoSampleMR (v. 0.5.0) [34,35]. In order to assess potential bidirectional pathways, we conducted two-sample MR analyses with cEF as the exposure for one direction, and as the outcome in the other direction (figure 1), when assessing causal relationships with liability to schizophrenia, MDD, anxiety, smoking initiation, drinks per week, alcohol dependence and CUD.

We used independent genome-wide significant SNPs for the exposure of interest as instruments in the MR analyses, except when anxiety, alcohol dependence and CUD were the exposures, where we used a *p*-value threshold of 1 × 10^−05^ to select SNPs due to the low number of genome-wide significant SNPs. We excluded all SNPs in linkage disequilibrium (LD) using an *r*^2^ of 0.001, a window of 10 000 kb and the European 1000 genomes reference panel. Where there were palindromic SNPs, we tried to infer the positive strand based on allele frequencies, but if this was not possible, then these SNPs were also excluded. Where an exposure SNP was not available in the outcome data, we attempted to identify a suitable proxy SNP using the LDproxy tool from LDlink [36]. We pruned the SNPs extracted to *r*^2^ ≥ 0.8 and extracted the first SNP that was present in the exposure and outcome data. After exclusions and identifying any proxy SNPs, we searched for the remaining cEF SNPs in the outcome GWAS (73 for schizophrenia, 85 for MDD, 83 for anxiety, 82 for smoking initiation, 83 for drinks per week, 73 for alcohol dependence and 73 for CUD) and the remaining mental health and substance use exposure SNPs in the cEF outcome (175 for schizophrenia, 29 for MDD, 17 for anxiety, 187 for smoking initiation, 67 for drinks per week, 19 for alcohol dependence and 37 for CUD). The GWAS summary statistics for the exposure and outcome in each analysis were harmonized so that the SNP allele-exposure and SNP allele-outcome associations were in the same direction.

We used several different MR methods to assess these putative causal relationships: inverse-variance weighted (IVW) [37], MR-Egger [38], weighted median [39], simple mode and weighted mode [40] and Steiger filtering [34] MR methods. We used the IVW approach as our main method with the other methods used as sensitivity analyses.

The IVW approach constrains the intercept to pass through zero, assuming no horizontal pleiotropy. We tested for heterogeneity between the individual SNPs included in the genetic instrument using Cochran's test of heterogeneity. The MR-Egger method tests for overall directional pleiotropy by not constraining the intercept to pass through zero. If the intercept is not zero then this is indicative of directional horizontal pleiotropy. We also assessed heterogeneity between the individual SNPs while adjusting for any directional pleiotropy for the MR-Egger method using Rucker's Q test. We used the weighted median method to obtain estimates under the assumption that at least 50% of the SNPs satisfy the MR assumptions and are valid IVs. Finally, we used the mode-based approaches to obtain estimates for the largest cluster of SNPs, where SNPs not in that cluster could be invalid. The weighted method accounts for the largest weights of SNPs. We also conducted single SNP and leave-one-out analyses.

Where we found evidence of a bidirectional causal relationship, we ran Steiger filtering. This allows orientation of the direction of effect where the underlying biology of genetic variants is less clear, by identifying which SNPs explain more variance in the outcome than the exposure and then repeating the MR analyses excluding those SNPs to rule out reverse causation [34].

In cases where we found evidence for a causal effect between a given exposure and outcome, we present plots of these results in the electronic supplementary material. These plots include scatter plots of the SNP-exposure and SNP-outcome associations with the causal effect estimates from each MR method presented, forest plots for causal effects of each SNP in the instrument (which can indicate if heterogeneity is present), plots presenting the leave-one-out results and funnel plots of each SNP included in the instrument, where a symmetrical plot indicates that the effects of each SNP included are similar to the average effect and asymmetry may indicate horizontal pleiotropy.

We calculated weighted and unweighted regression dilution I-squared statistics for each analysis [41], presented in the electronic supplementary material, table S1, which give an indication of the amount of bias in the ‘NO Measurement Error’ (NOME) assumption in the MR-Egger estimate [39]. If the I-squared statistic is 0.9 or above, this indicates minimal bias in the MR-Egger estimate, and therefore, we present the MR-Egger results for these associations. If either the weighted or unweighted I-squared statistics were between 0.6 and 0.9, this may indicate regression dilution bias, and therefore, we ran simulation extrapolation (SIMEX) corrections, to obtain bias-adjusted point estimates for MR-Egger and we present these results in place of MR-Egger. Anything below 0.6 means that the bias may be too large, and therefore, we do not report either the SIMEX correction or the MR-Egger results. We also estimated the mean F-statistic for each analysis, indicative of instrument strength, where a value under 10 may indicate a weak instrument [41].

## 3. Results

Our two-sample MR results for the causal effects of cEF on mental health and substance use outcomes are presented in table 2.

**Table 2.** Two-sample Mendelian randomization results with executive function (cEF) as the exposure.

outcome	method	NSNPs	OR or beta (95% CI)	*p*-value	heterogeneity test *p*-value	directional pleiotropy intercept (95% CI; *p*-value)
schizophrenia	IVW	73	0.10 (0.05, 0.19)	3.43 × 10^−12^	3.52 × 10^−61^	
	MR Egger^a^	—	—	—	—	—
	weighted median	73	0.15 (0.09, 0.24)	1.12 × 10^−15^		
	simple mode	73	0.11 (0.04, 0.33)	2.04 × 10^−04^		
	weighted mode	73	0.12 (0.05, 0.29)	1.53 × 10^−05^		
MDD	IVW	85	0.52 (0.38, 0.72)	5.23 × 10^−05^	8.22 × 10^−03^	
	MR Egger^a^	—	—	—	—	—
	weighted median	85	0.45 (0.30, 0.67)	1.12 × 10^−04^		
	simple mode	85	0.36 (0.13, 1.10)	0.06		
	weighted mode	85	0.33 (0.12, 0.96)	0.05		
anxiety	IVW	83	0.49 (0.19, 1.23)	0.13	0.09	
	MR Egger^b^	83	0.39 (3.67 × 10^−4^, 420.28)	0.79		0.002 (−0.05, 0.06; 0.94)
	weighted median	83	0.31 (0.09, 1.07)	0.07		
	simple mode	83	0.09 (0.004, 2.03)	0.14		
	weighted mode	83	0.16 (0.009, 2.99)	0.23		
smoking initiation	IVW	82	0.87 (0.74, 1.02)	0.09	2.64 × 10^−67^	
	MR Egger^a^	—	—	—	—	—
	weighted median	82	0.88 (0.78,0.99)	0.04		
	simple mode	82	0.81 (0.51,1.29)	0.39		
	weighted mode	82	0.81 (0.49, 1.37)	0.44		
drinks per week	IVW	83	−0.06 (−0.10, −0.02)	0.003	8.81 × 10^−26^	
	MR Egger^a^	—	—	—	—	—
	weighted median	83	−0.05 (−0.08, −0.01)	0.008		
	simple mode	83	−0.05 (−0.14, 0.04)	0.29		
	weighted mode	83	−0.04 (−0.12, 0.04)	0.33		
alcohol dependence	IVW	73	0.50 (0.23, 1.09)	0.08	0.17	
	MR Egger^a^	—	—	—	—	—
	weighted median	73	0.37 (0.12, 1.14)	0.92		
	simple mode	73	0.33 (0.02, 5.61)	0.44		
	weighted mode	73	0.47 (−0.04, 5.36)	0.55		
CUD	IVW	73	0.27 (0.12, 0.61)	1.58 × 10^−03^	6.17 × 10^−09^	
	MR Egger^a^	—	—	—	—	—
	weighted median	73	0.14 (0.06, 0.35)	1.84 × 10^−05^		
	simple mode	73	0.05 (0.007, 0.38)	4.56 × 10^−03^		
	weighted mode	73	0.08 (0.01, 0.47)	7.19 × 10^−03^		

^a^NOME assumption violated for MR-Egger and value below 0.6; therefore, no results presented.

^b^I-squared value between 0.6 and 0.9 so SIMEX correction is presented instead of MR-Egger. MDD = major depressive disorder, CUD = cannabis use disorder, OR-odds ratio, CI = confidence interval, IVW = inverse-variance weighted, MR = Mendelian randomization, SNP = single-nucleotide polymorphism.

### 3.1. Schizophrenia

We found strong evidence of a causal effect of increased cEF on reduced odds of schizophrenia (IVW: OR = 0.10; 95% CI 0.05 to 0.19; *p*-value = 3.43 × 10^−12^) for all methods except MR Egger, which we were unable to estimate due to violation of the NOME assumption (see electronic supplementary material, table S1). These results were in a consistent direction across the different MR analyses (electronic supplementary material, figure S1). However, we did observe evidence of heterogeneity for the IVW estimate (electronic supplementary material, figure S2) and some asymmetry in the funnel plot (electronic supplementary material, figure S3), although our leave-one-out analyses did not indicate that a single SNP was driving the association (electronic supplementary material, figure S4). Steiger filtering indicated that only 41% of SNPs instrumenting cEF explained more variance in cEF than schizophrenia, and results were attenuated when repeating analyses with this subset of SNPs. However, these results were in the same direction as the main results (electronic supplementary material, table S2).

### 3.2. Major depressive disorder

We found strong evidence of a causal effect of increased cEF on reduced odds of MDD (IVW: OR = 0.52; 95% CI 0.38 to 0.72; *p*-value = 5.23 × 10^−05^). These results were in a consistent direction across the different MR analyses (electronic supplementary material, figure S5), and there was evidence of a causal effect for all methods except simple mode and MR Egger, which again we were unable to estimate due to violation of the NOME assumption. Here we also found evidence of heterogeneity for the IVW estimate (electronic supplementary material, figure S6) and some asymmetry in the funnel plot (electronic supplementary material, figure S7); however, leave-one-out analyses did not indicate that a single SNP was driving the association (electronic supplementary material, figure S8). Steiger filtering indicated that 81% of SNPs instrumenting cEF explained more variance in cEF than MDD, but again results were similar to the main results when using this subset of SNPs (electronic supplementary material, table S2).

### 3.3. Anxiety

We did not find evidence of a causal effect of cEF on anxiety liability (IVW: OR = 0.49; 95% CI 0.19 to 1.23; *p*-value = 0.13).

### 3.4. Smoking initiation

We did not find clear evidence of a causal effect of cEF on smoking initiation (IVW: OR = 0.87; 95% CI 0.74 to 1.02; *p*-value = 0.09) for any of the MR analyses.

### 3.5. Drinks per week

We found some evidence of a causal effect of increased cEF on decreased number of alcoholic drinks per week consumed (IVW: *β* = −0.06; 95% CI −0.10 to −0.02; *p*-value = 0.003). These results were in a consistent direction across the different MR analyses (electronic supplementary material, figure S9), although there was only evidence of a causal effect for the IVW method. We also found evidence of heterogeneity here (electronic supplementary material, figure S10) and slight asymmetry in the funnel plot (electronic supplementary material, figure S11); however, leave-one-out analyses did not indicate that a single SNP was driving the association (electronic supplementary material, figure S12).

### 3.6. Alcohol dependence

We did not find clear evidence of a causal effect of cEF on alcohol dependence liability (IVW: OR = 0.50; 95% CI 0.23 to 1.28; *p*-value = 0.08) for any of the MR analyses.

### 3.7. Cannabis use disorder

We found strong evidence of a causal effect of increased cEF on reduced odds of CUD (IVW: OR = 0.27; 95% CI 0.12 to 0.61; *p*-value = 1.58 × 10^−03^) for all methods except MR Egger, which we were unable to estimate due to violation of the NOME assumption (see electronic supplementary material, table S1). These results were in a consistent direction across the different MR analyses (electronic supplementary material, figure S13). However, we did observe evidence of heterogeneity for the IVW estimate (electronic supplementary material, figure S14) and slight asymmetry in the funnel plot (electronic supplementary material, figure S15), although our leave-one-out analyses did not indicate that a single SNP was driving the association (electronic supplementary material, figure S16).

Our two-sample MR results for causal effects of the mental health and substance use phenotypes on cEF are presented in table 3.

**Table 3.** Two-sample Mendelian randomization results with executive function (cEF) as the outcome.

exposure	method	NSNPs	beta (95% CI)	*p*-value	heterogeneity test *p*-value	directional pleiotropy intercept (95% CI; *p*-value)
schizophrenia	IVW	175	−0.04 (−0.04, −0.03)	3.25 × 10^−27^	3.04 × 10^−72^	
	MR Egger^b^	175	−0.05 (−0.07, −0.03)	5.77 × 10^−05^		9.0 × 10^−04^ (−6.13 × 10^−04^, 0.002; 0.24)
	weighted median	175	−0.03 (−0.03, −0.02)	8.08 × 10^−21^		
	simple mode	175	−0.04 (−0.06, −0.01)	2.14 × 10^−03^		
	weighted mode	175	−0.04 (−0.06, −0.02)	7.93 × 10^−04^		
MDD	IVW	29	−0.02 (−0.05, 0.01)	0.31	3.45 × 10^−19^	
	MR Egger^a^	—	—	—	—	—
	weighted median	29	−0.03 (−0.05, −0.01)	0.01		
	simple mode	29	−0.04 (−0.08, −0.006)	0.03		
	weighted mode	29	−0.03 (−0.06, −0.002)	0.04		
anxiety	IVW	17	8.69 × 10^−04^ (−0.003, 0.005)	0.68	0.17	
	MR Egger^a^	—	—	—	—	—
	weighted median	17	7.19 × 10^−04^ (−0.004, 0.006)	0.78		
	simple mode	17	0.004 (−0.007, 0.01	0.49		
	weighted mode	17	0.002 (−0.007, 0.01)	0.67		
smoking initiation	IVW	187	−0.06 (−0.09, −0.03)	6.11 × 10^−05^	4.23 × 10^−88^	
	MR Egger^a^	—	—	—	—	—
	weighted median	187	−0.04 (−0.07, −0.01)	2.61 × 10^−03^		
	simple mode	187	−0.01(−0.11, 0.08	0.80		
	weighted mode	187	−0.01(−0.12, 0.09)	0.82		
drinks per week	IVW	67	−0.007 (−0.17, 0.16)	0.93	1.09 × 10^−51^	
	MR Egger^a^	—	—	—	—	—
	weighted median	67	−0.05 (−0.16, 0.07	0.45		
	simple mode	67	−0.10 (−0.31, 0.11)	0.34		
	weighted mode	67	−0.05 (−0.15, 0.06)	0.41		
alcohol dependence	IVW	19	−3.31 × 10^−03^ (−8.21 × 10^−03^, 1.65 × 10^−03^)	0.19	0.08	1.93 × 10^−05^ (−1.99 × 10^−03^, 2.03 × 10^−03^; 0.99)
	MR Egger^b^	19	−3.74 × 10^−03^ (−0.01, 3.66 × 10^−03)^	0.33		
	weighted median	19	−3.67 × 10^−03^ (−9.78 × 10^−03^, 2.44 × 10^−03^)	0.24		
	simple mode	19	−3.69 × 10^−03^ (−0.01, 7.25 × 10^−03^)	0.52		
	weighted mode	19	−4.28 × 10^−03^ (−0.01, 3.58 × 10^−03^)	0.30		
CUD	IVW	37	−6.42 × 10^−03^ (−0.01, −9.56 × 10^−04^)	0.09	1.71 × 10^−08^	
	MR Egger^a^	—	—	—	—	—
	weighted median	37	−5.16 × 10^−03^ (−0.01, 1.98 × 10^−03^)	0.16		
	simple mode	37	−4.65 × 10^−03^ (−0.02, 9.96 × 10^−03^)	0.54		
	weighted mode	37	−3.65 × 10^−03^ (−0.02, 0.01)	0.61		

^a^NOME assumption violated for MR-Egger and value below 0.6; therefore, no results presented.

^b^I-squared value between 0.6 and 0.9 so SIMEX correction is presented instead of MR-Egger. MDD = major depressive disorder, CUD = cannabis use disorder, OR-odds ratio, CI = confidence interval, IVW = inverse-variance weighted, MR = Mendelian randomization, SNP = single-nucleotide polymorphism.

### 3.8. Schizophrenia

We found strong evidence of a causal effect of increased odds of schizophrenia on decreased cEF (IVW: *β* = −0.04; 95% CI −0.04 to −0.03; *p*-value = 3.25 × 10^−27^). These results were in a consistent direction across the different MR analyses (electronic supplementary material, figure S17). However, we did observe evidence of heterogeneity for the IVW estimate (electronic supplementary material, figure S18) and some asymmetry in the funnel plot (electronic supplementary material, figure S19), although there was little evidence of directional pleiotropy from the SIMEX estimate and our leave-one-out analyses did not indicate that a single SNP was driving the association (electronic supplementary material, figure S20). Steiger filtering indicated that all SNPs instrumenting schizophrenia explained more variance in schizophrenia than cEF; therefore, these analyses were not repeated (electronic supplementary material, table S2).

### 3.9. Major depressive disorder

We did not find evidence of a causal effect of MDD liability on cEF (IVW: *β* = −0.02; 95% CI −0.05 to 0.01; *p*-value = 0.31) using IVW. However, evidence was stronger using other sensitivity methods and the direction of effect was consistent across all approaches i.e. for increased odds of MDD on decreased cEF. Steiger filtering indicated that all SNPs instrumenting schizophrenia explained more variance in MDD than cEF; therefore, these analyses were not repeated (electronic supplementary material, table S2).

### 3.10. Anxiety

We did not find evidence of a causal effect of anxiety liability on cEF (IVW: *β* = 8.69 × 10^−04^; 95% CI −0.003 to 0.005; *p*-value = 0.68) for any of the MR analyses.

### 3.11. Smoking initiation

We found strong evidence of a causal effect of smoking initiation on decreased cEF (IVW: *β* = −0.06; 95% CI −0.09 to −0.03; *p*-value = 6.11 × 10^−05^), and there was evidence of this causal effect using the weighted median method but not the other MR methods, although the direction of effect was consistent (electronic supplementary material, figure S21). We did find evidence of heterogeneity for the IVW estimate (electronic supplementary material, figure S22) and slight asymmetry in the funnel plot (electronic supplementary material, figure S23); however, our leave-one-out analyses did not indicate that a single SNP was driving the association (electronic supplementary material, figure S24).

### 3.12. Drinks per week

We did not find clear evidence of a causal effect of cEF on number of alcoholic drinks per week consumed (IVW: *β* = −0.007; 95% CI −0.17 to 0.16; *p*-value = 0.93).

### 3.13. Alcohol dependence

We did not find clear evidence of a causal effect of alcohol dependence liability on cEF (IVW: *β* = −3.31 × 10^−03^; 95% CI −8.21 × 10^−03^ to 1.65 × 10^−03^; *p*-value = 0.09) for any of the MR analyses.

### 3.14. Cannabis use disorder

We did not find clear evidence of a causal effect of CUD liability on cEF (IVW: *β* = −6.42 × 10^−03^; 95% CI −0.01 to 9.56 × 10^−04^; *p*-value = 0.09) for any of the MR analyses.

## 4. Discussion

We examined whether there was evidence of causal effects of cEF on schizophrenia, MDD, anxiety, smoking initiation, drinks per week, alcohol dependence and CUD. We also examined the reverse direction (i.e. causal effects of mental health and substance use on cEF). Evidence of a causal effect in both directions may be indicative of a bidirectional relationship or some other underlying common risk factor.

Our main findings were evidence of a causal relationship between increased cEF and reduced schizophrenia liability in both directions. Steiger filtering supported the finding of bidirectional effects, despite some attenuation of results for cEF on schizophrenia. This causal effect supports previous observational studies, which have found that people with schizophrenia have poorer EF in all subdomains compared with controls [1,4–6], although the direction of effect is unclear. However, our MR analyses additionally provide evidence that these associations may reflect causal pathways. The fact that we find causal effects between schizophrenia liability and cEF in both directions may point to this association being bidirectional or due to an underlying common risk factor.

The observed causal effect of increased cEF on reduced MDD liability is also interesting, as previous studies have provided mixed evidence for the directionality of this relationship. For example, it has previously been found that EF deficits are experienced by those with a diagnosis of depression even when not currently experiencing depressive symptoms [7] and that this association is a function of characteristics of depression that vary by individual [8]. Our results extend and support these findings and suggest that this association may predominantly be due to potential causal effects of cEF on MDD. We did not find strong evidence of a causal effect of MDD on cEF using the IVW approach; however, evidence was stronger when using other MR methods, and the direction of effect we observed was negative and consistent across these. It may also be possible that there is lower power in the MDD instrument than the cEF instrument, due to the lower number of SNPs, which may also be why we do not observe a consistent effect of MDD on cEF. Therefore, we cannot rule out the possibility of a bidirectional relationship and Steiger filtering suggested that the effects could be bidirectional as well. Therefore, future studies should examine the possibility of bidirectional relationships further.

We cannot draw any strong conclusions from our results regarding anxiety and cEF. While we do not find evidence of a causal relationship, this does not mean that there is definitely no effect, but rather that our study may have lacked the power to detect a causal effect here, particularly given that we find some evidence of a possible causal effect of MDD on cEF in our sensitivity analyses and previous studies have reported high genetic correlations between MDD and anxiety [42]. Thus, the association found in previous studies needs further investigation still [9,10].

Finally, we also found some evidence of a causal effect of smoking initiation on decreased cEF and increased cEF on decreased drinks per week and reduced CUD liability, all in a consistent direction with previous observational studies [11–15]. However, evidence for the smoking and drinks per week findings was weak, so further studies examining this would be useful.

Our results suggest that there are possible causal associations between cEF and mental health/substance use phenotypes. We see some evidence of increased cEF leading to decreased risk of mental health outcomes and CUD, suggesting that early interventions aiming to improve EF, e.g. through teaching compensatory strategies, could improve outcomes in these areas. Our finding of potentially bidirectional causal effects suggests that the relationship is not straightforward, and that mental health may also impact EF, for example in individuals with schizophrenia it may be the case that poorer EF is involved in the development of schizophrenia, but that having schizophrenia also results in abnormalities in frontal regions e.g. decreased grey matter, that may disrupt EF [43,44]. Therefore, while interventions to improve EF may decrease the risk of developing mental health conditions, it would also be useful to target such interventions to improve EF for those who already have mental health conditions, like schizophrenia, where difficulties in EF may be more prevalent.

Our results are in contrast with a previous study which used latent causal variable (LCV) analyses and did not find evidence of any causal effects of cEF on schizophrenia, MDD, anxiety, alcohol use disorder or other traits examined [20]. LCV analysis relies on the assumption that there is a latent variable that mediates the genetic correlation between two traits and uses whole genome summary statistics to estimate genetic causality. One trait is partially genetically causal for the other if it is strongly genetically correlated with the LCV. However, LCV aims to capture the overall direction of causality and, therefore, may be less appropriate for relationships where a bidirectional effect may be present, as we observe in our study, whereas MR does allow for bidirectional relationships [45]. LCV has better control for pleiotropy [20], meaning the difference between our results and those in the previous LCV analysis may reflect the presence of pleiotropy, which we were unable to directly test for in several of our analyses. We were able to test this for our MR of schizophrenia on cEF and found no evidence of pleiotropy. However, this should be considered when interpreting our other results.

In addition, while the original GWAS paper for this cEF measure demonstrated that despite having many working memory measures included in the model it predicted cEF well, it is important still to note that there may be aspects of cEF that are less captured by this measure.

### 4.1. Limitations

There are a number of limitations to our study that should be considered when interpreting these results. First, there could be low statistical power to detect causal effects for some of the analyses. In particular, where anxiety, alcohol dependence and CUD are the exposures, there were a low number of genome-wide significant SNPs. To overcome this issue, we lowered the *p*-value threshold for our MR analyses to 1 × 10^−5^, but this means that any interpretation of these results should be approached with caution and revisiting this causal relationship when larger GWAS are available would be valuable. Second, in the majority of our analyses (i.e. in both directions for schizophrenia, MDD, smoking initiation, drinks per week and CUD), evidence of heterogeneity was observed in the IVW estimates, which could suggest that horizontal pleiotropy is present (e.g. that independent pathways are responsible for the influence of SNPs on the exposure and outcome). Therefore, caution should be used when interpreting these results. However, we did test for violations of other MR assumptions using additional MR sensitivity analyses and the direction of the results was consistent with the direction of the main results. Despite this, in a number of our analyses we could not test for directional pleiotropy due to the I-squared estimate being too low.

Third, MR is also subject to some general limitations [46]. For example, the ‘Winner's curse’ can occur when the SNPs used are based on the discovery GWAS only (as opposed to combined discovery and replication results), meaning that SNP-trait effects may be overestimated. This can mean that the MR estimate is biased towards the null. Thus, the focus of our results is on the direction of effect as opposed to the size of any causal effects, although the latter may still be somewhat informative.

Some further limitations, which are relevant to any study drawing on GWAS of psychiatric phenotypes, are that psychiatric phenotypes are highly heterogeneous in terms of symptomology, comorbidities and possible risk factors, and often rely on binary measures which are limited for phenotypes which are really extremes of continuous underlying traits. These limitations in the context of MR are described further by Wootton *et al*. [47] and highlight the need to be cautious when interpreting our findings.

## 5. Conclusion

Our findings suggest a bidirectional causal relationship between cEF and schizophrenia liability, where increased schizophrenia liability is associated with decreased cEF and vice versa, as well as causal effects of increased cEF on reduced MDD liability and CUD liability and decreased drinks per week, and a causal effect of smoking initiation on decreased cEF. These results require further study to better understand the mechanisms behind these causal effects. Future research would benefit from better powered GWAS for anxiety where a lack of power may explain why we did not detect any causal effects. Our results may inform prioritization of experimental medicine studies (e.g. of interventions targeting EF) to improve the likelihood of successful translation and suggest that these interventions may be useful prior to the onset of schizophrenia and MDD in particular.

## Data Availability

The GWAS data for cEF will be made available on the GWAS catalogue and is currently available on request from the Friedman Lab at University of Colorado, Boulder (naomi.friedman@colorado.edu). Once the paper associated with this GWAS has been published, the data will be made publicly available for download without restriction. Other GWAS data used in this study are publicly available GWAS data for schizophrenia (https://www.med.unc.edu/pgc/download-results/scz/), MDD (https://www.med.unc.edu/pgc/download-results/mdd/), anxiety (https://www.med.unc.edu/pgc/download-results/angst/?choice=Other+GWAS+DataAnxiety+Neuro+Genetics+Study+%28ANGST%29), alcohol dependence (https://figshare.com/articles/dataset/sud2018-alc/14672187) and CUD (https://figshare.com/articles/dataset/sud2020-cud/14842692). Finally, GWAS data for smoking initiation and drinks per week with UK Biobank and 23andMe removed can be found here: https://conservancy.umn.edu/handle/11299/201564. The full GWAS summary statistics for the 23andMe discovery data set (which we combined with the smoking initiation and drinks per week publicly available data) will be made available through 23andMe to qualified researchers under an agreement with 23andMe that protects the privacy of the 23andMe participants. Please visit https://research.23andme.com/collaborate/#dataset-access/ for more information and to apply to access the data. Code availability: The analysis code that forms the basis of the results presented here is available from the University of Bristol's Research Data Repository, data.bris, at https://doi.org/10.5523/bris.2ejxinnlt7kmr2hda2infx5r1k [48]. The data are provided in the electronic supplementary material [49].
